# Disseminated HIV-Associated Burkitt Lymphoma With Cardiac and Presumed Central Nervous System Involvement

**DOI:** 10.1016/j.jaccas.2026.110281

**Published:** 2026-09-11

**Authors:** Daniel Tsai, Marina Trilesskaya

**Affiliations:** a Department of Internal Medicine, Highland Hospital, Alameda Health System, Oakland, California, USA; b Department of Cardiology, Highland Hospital, Alameda Health System, Oakland, California, USA

**Keywords:** cancer, echocardiography, imaging

## Abstract

**Background:**

Cardiac involvement in Burkitt lymphoma (BL) is rare, with fewer than 30 cases reported in the literature. Contrast echocardiography can characterize intracardiac masses in resource-limited settings, although multimodal imaging is paramount.

**Case Summary:**

A 52-year-old man with HIV presented with axillary lymphadenopathy, constitutional symptoms, and a left oculomotor nerve palsy. Imaging revealed splenic lesions and a right atrial mass. Contrast echocardiography demonstrated vascularity of the mass with invasion into the crux. Cardiac magnetic resonance imaging confirmed involvement of the atrial and ventricular septum. He was diagnosed with Ann Arbor stage 4 HIV-associated BL and started on R-hyperCVAD, with resolution of the cardiac mass after 2 months of treatment.

**Discussion:**

This is a rare case of disseminated BL with combined atrial and ventricular septal involvement, not previously reported.

**Take-Home Message:**

Early multimodal cardiac imaging in disseminated BL disease is important regardless of presenting cardiac symptoms.

## Visual Summary

**Figure:**
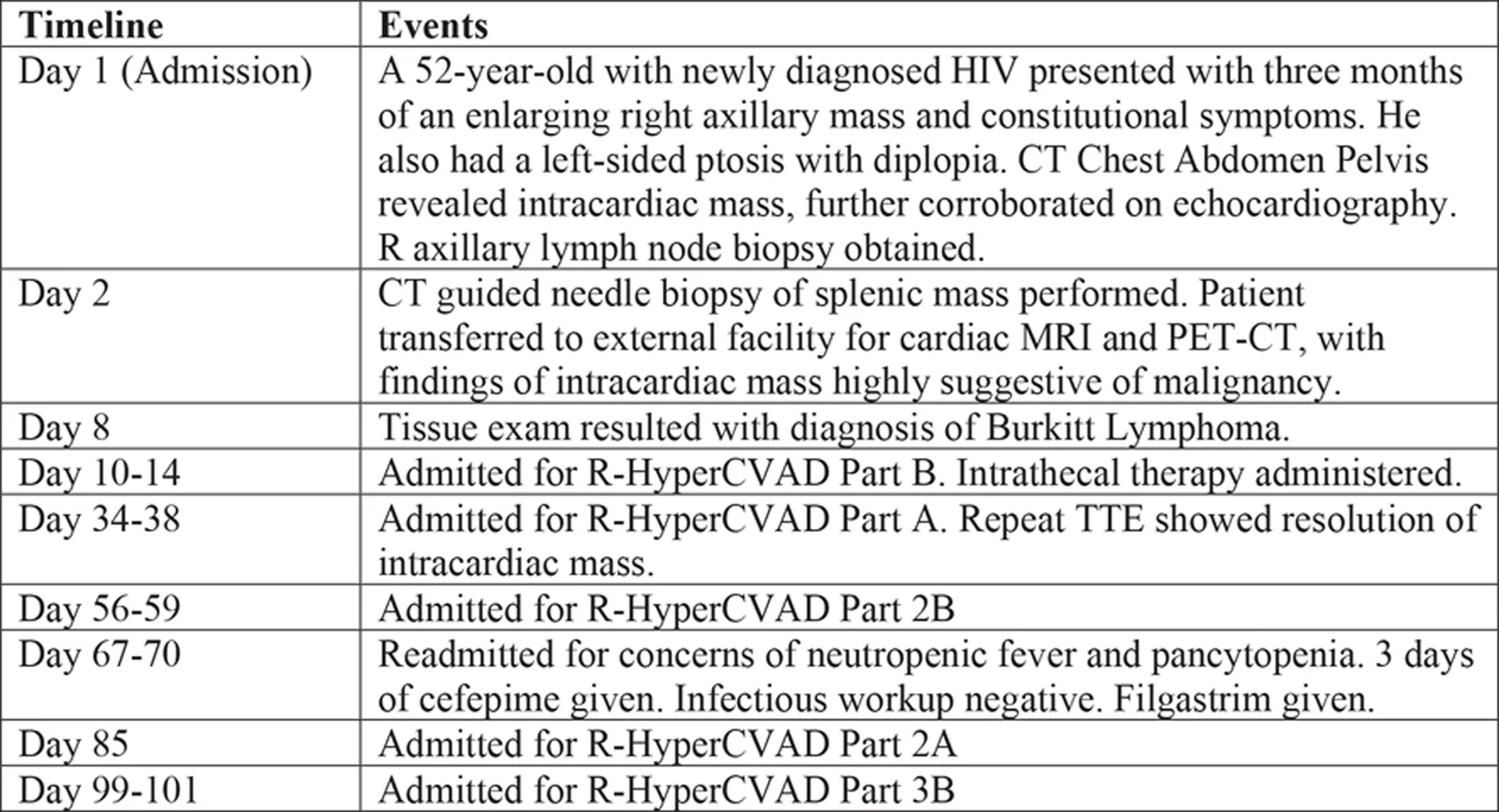

Burkitt lymphoma (BL) is a highly aggressive B-cell non-Hodgkin lymphoma (NHL) characterized by chromosomal translocation t (8; 14) and constitutive activation of the myelocytomatosis oncogene. BL accounts for <1% of NHL in adults, with higher incidences among immunocompromised subjects, such as patients with HIV and organ transplantation recipients.^1^^,^^2^ Although BL classically presents with rapidly enlarging tumor masses in the facial bones, jaw or mandible, and/or abdominal organs, cardiac involvement is exceedingly rare.

Diagnosis of cardiac involvement is often delayed because of nonspecific or absent symptoms, often conferring a worse prognosis.^3^, ^4^, ^5^, ^6^ In resource-limited settings, contrast echocardiography can characterize the involved structures and vascularity of the mass. However, cardiac magnetic resonance imaging (MRI) is better at soft-tissue contrast for mass characterization, whereas positron emission tomography-computed tomography (PET-CT) offers metabolic assessment and is crucial for staging and evaluating the risk of myocardial infiltration before chemotherapy.^3^^,^^6^

We report a case of disseminated HIV associated BL with progressive lymphadenopathy and involvement of the spleen, heart, and presumed central nervous system. In addition to the rarity of BL with cardiac involvement, this case highlights the diagnostic challenges and treatment considerations specific to intracardiac disease.

## History of presentation

A 52-year-old with newly diagnosed HIV on antiretroviral therapy presented with 3 months of an enlarging right axillary mass, night sweats, unintentional weight loss, and debilitating fatigue. He concurrently developed a left-sided ptosis with diplopia. The symptoms raised concern for a hematologic malignancy, prompting expedited workup. He denied shortness of breath, palpitations, lightheadedness, or chest pain throughout his course. On admission, he was hemodynamically stable. Physical exam revealed an enlarged right axillary and supraclavicular lymph node and a left cranial nerve 3 palsy.

## Past Medical History

Newly diagnosed HIV (cluster of differentiation [CD] 4 + count 224 cells/uL at diagnosis) on antiretroviral therapy. No prior cardiac history.

## Differential Diagnosis

Given incidental discovery of a right atrial mass in a patient with disseminated lymphoma, the differentials include intracardiac thrombus, vegetation, primary cardiac tumor (eg, myxoma, sarcoma), and secondary cardiac involvement from the highly suspected lymphoma upon presentation. The vascularity of the mass on contrast echocardiography and hypermetabolic activity on PET-CT favored a malignant lymphomatous process over thrombus or vegetation.

## Investigations

Labs demonstrated an elevated lactate dehydrogenase of 422 U/L and uric acid of 9.8 mg/dL, with normal natriuretic peptides, troponin, complete blood count, comprehensive metabolic panel, and coagulation studies. Colony-stimulating factor analysis showed an elevated opening pressure of 30 cm H_2_O but was otherwise bland and acellular, precluding cytologic analysis.

Contrast-enhanced CT of the chest, abdomen, and pelvis revealed right hilar lymphadenopathy, pulmonary nodules, multiple splenic lesions—the largest measuring 5.5 cm—and a hypodense structure in the right atrium (Figure 1). Brain MRI demonstrated a subtle T2 fluid-attenuated inversion recovery hyperintensity in the left cavernous sinus with extension across the midline posteriorly. PET-CT revealed widespread hypermetabolic disease involving the right atrial mass, pulmonary nodules, right axillary and chest wall masses, left adrenal mass, and multiple lymph nodes above and below the diaphragm.

**Figure 1:**
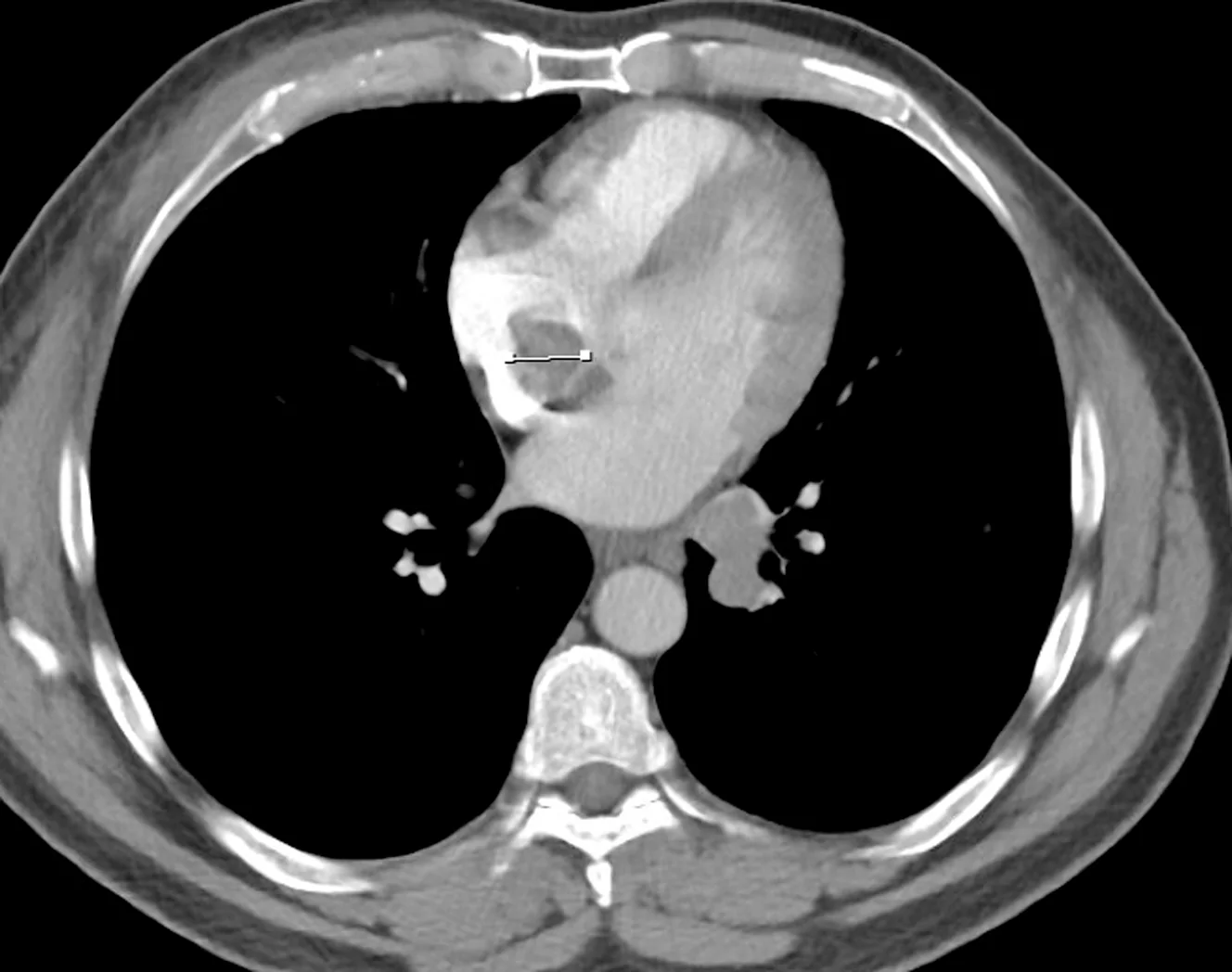
Hyperlucent Structure in the Right Atrium Contrast-enhanced computed tomography of the chest showing a hypodense structure within the right atrium, an incidental finding identified during staging workup for systemic lymphadenopathy. This finding prompted further echocardiographic evaluation for an intracardiac mass.

Baseline transthoracic echocardiogram identified a 2.5-cm mass attached to the interatrial septum (Figure 2, Video 1). Transesophageal echocardiogram further characterized the mass as large with an anechoic center concerning for necrosis. Given limited advanced imaging resources at a safety-net hospital, contrast was used to further characterize the mass, confirming its vascularity and invasion into the crux (Figure 3). The patient was subsequently transferred to an external facility for cardiac MRI, which revealed a 2.3 × 2.3 × 2.6-cm mass attached to the atrial septum above the fossa ovalis without obstruction of the superior or inferior vena cava or the tricuspid valve, along with at least 2 additional tumor foci within the mid-to-apical ventricular septum (each measuring 1-2 cm). There was no definitive involvement of the ventricles, left atrium, or pericardial space. An occlusive right internal jugular vein thrombus was also noted, and anticoagulation was initiated.

**Figure 2:**
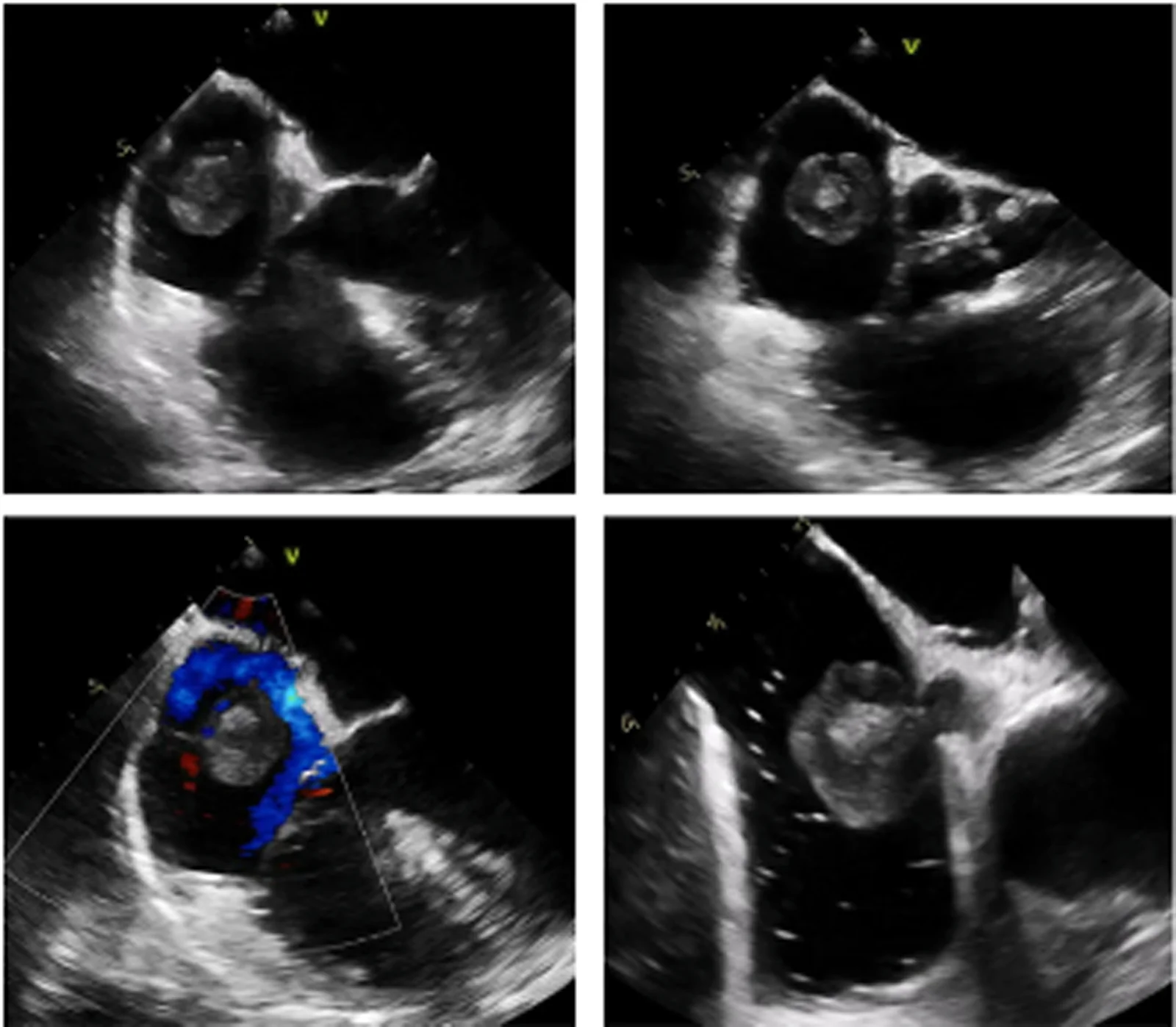
Mobile Circular Mass Attached to the Interatrial Septum, Demonstrated on Transthoracic Echocardiogram This finding was further characterized by transesophageal echocardiography with contrast and cardiac magnetic resonance imaging.

**Figure 3:**
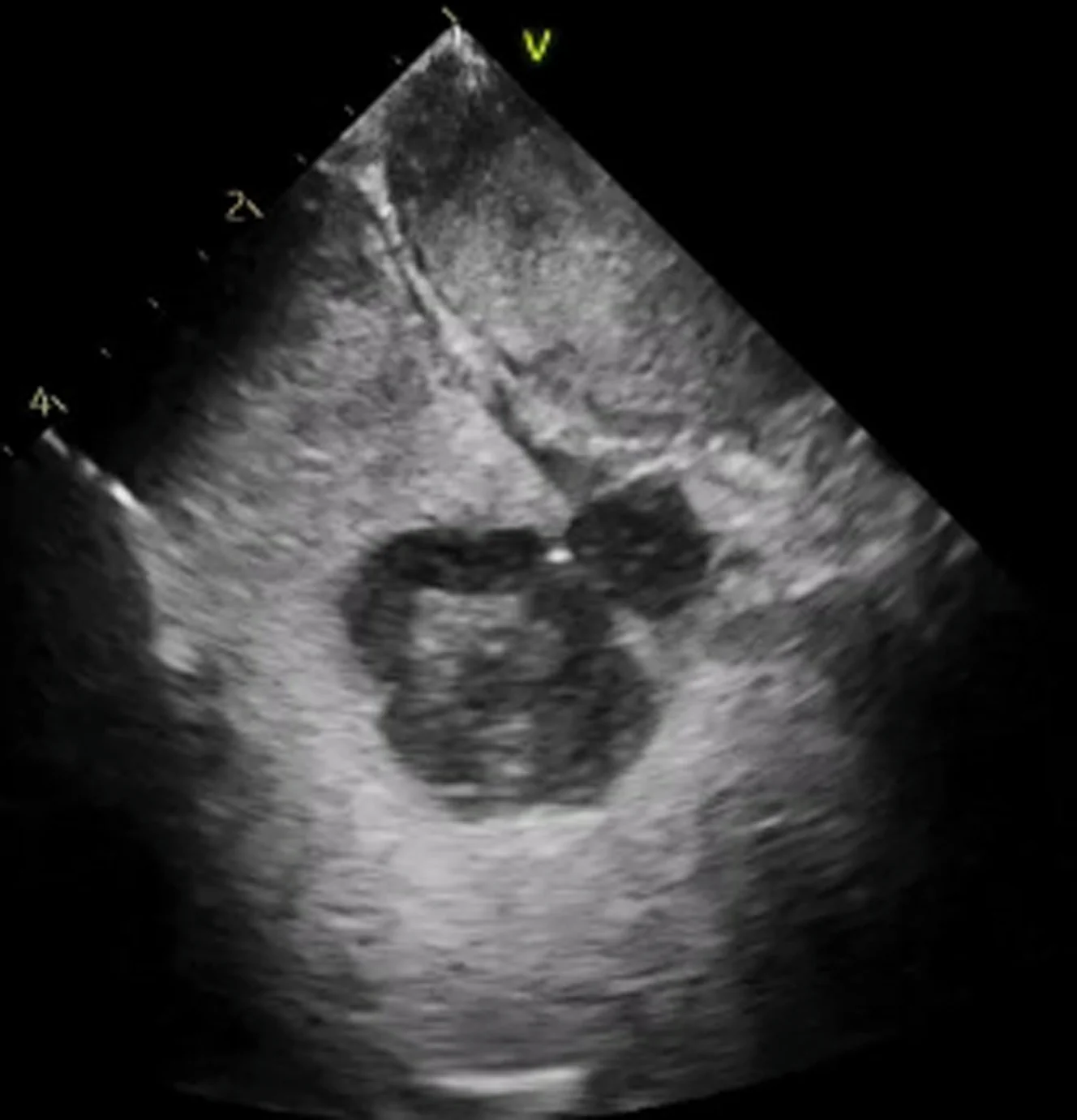
Contrast Further Characterizes the Mass as Invading the Crux of the Heart and Vascular Structures Contrast enhancement demonstrates perfusion within the mass, consistent with a vascular structure. In resource-limited settings, contrast echocardiography can provide diagnostically useful characterization of intracardiac masses when advanced imaging is not immediately available.

Core needle biopsy of the right axillary lymph node demonstrated diffuse atypical lymphocytes composed of medium-sized, monotonous cells with frequent mitoses, apoptotic bodies, and scattered tingible body macrophages, creating a classic starry sky pattern. Flow cytometry identified a monotypic CD10+/CD20+ B-cell lymphoma. Fluorescence in situ hybridization confirmed myelocytomatosis rearrangement. Splenic biopsy yielded similar findings, with immunohistochemistry demonstrating atypical lymphocytes expressing CD20, CD10, and a Ki-67 proliferation index approaching 100%.

## Management

The patient was diagnosed with Ann Arbor stage IV HIV-associated BL and started on rituximab plus hyperfractionated cyclophosphamide, vincristine, doxorubicin, and dexamethasone (R-hyperCVAD) therapy given in alternating cycles. Part A includes cyclophosphamide, vincristine, doxorubicin, rituximab, and dexamethasone. Part B includes high-dose methotrexate and intrathecal cytarabine and methotrexate for presumed central nervous system involvement.

## Outcome and Follow-Up

He underwent 2 complete cycles of R-HyperCVAD and is currently getting his third cycle. Complications included nausea from methotrexate and pancytopenia requiring granulocyte colony-stimulating factor and a 20% dose reduction of cytarabine. There was improvement in his cranial nerve 3 palsy, and the previously observed right atrial mass was no longer demonstrated on repeat transthoracic echocardiogram, consistent with resolution (Figure 4, Video 2). Total cumulative anthracycline dose at that time equated to 100 mg/m^2^.

**Figure 4:**
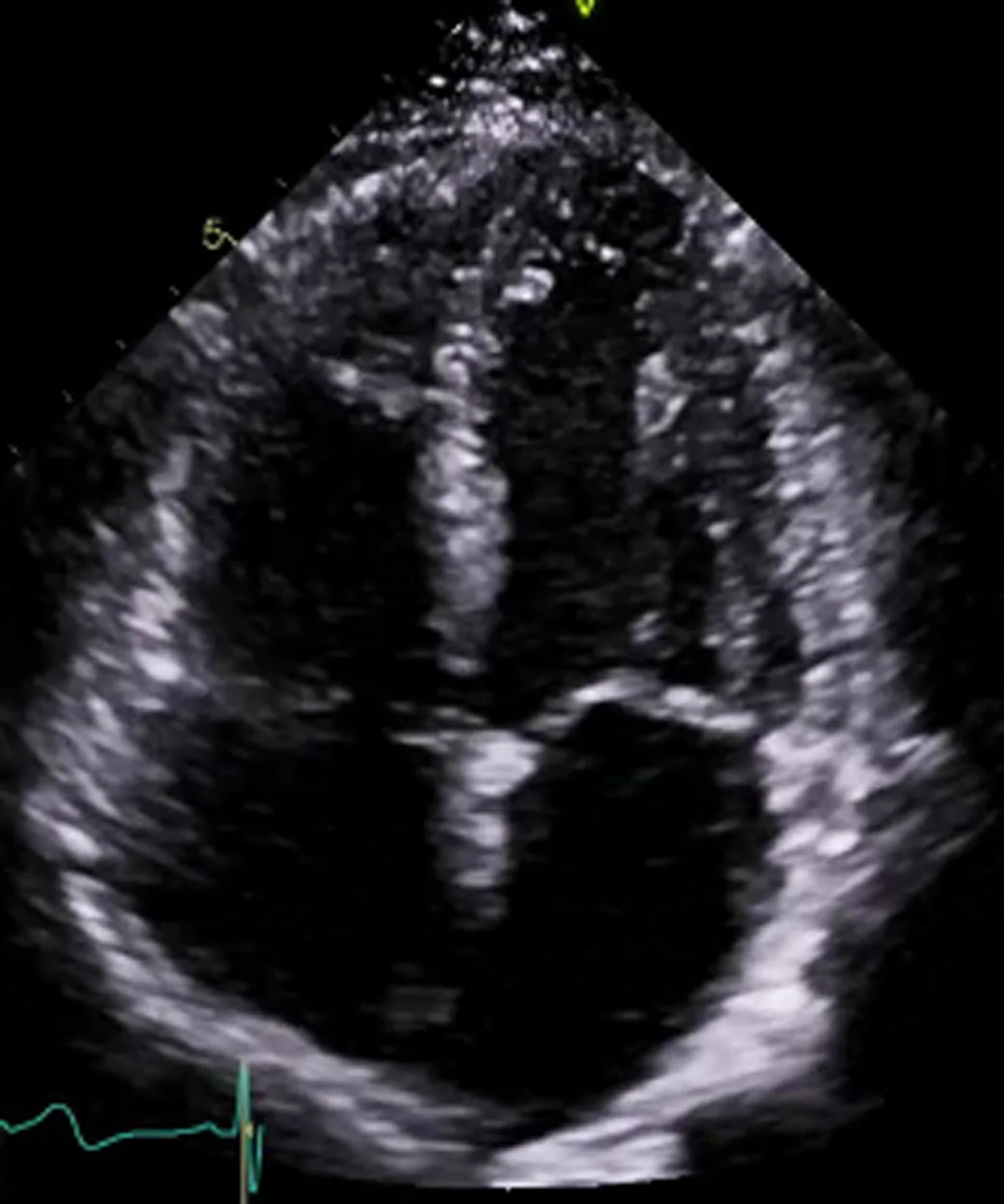
Resolution of the Previously Visualized Intracardiac Mass Follow-up transthoracic echocardiogram approximately 2 months after initiation of chemotherapy, demonstrating resolution of the previously visualized right atrial mass and response to systemic chemotherapy.

## Discussion

Cardiac involvement is rare in BL, with fewer than 30 cases documented in the literature.^7^ In a review of 22 reported cases, the median age was 38 years, with 41% of patients having underlying immunodeficiency. Presenting symptoms included palpitations, lightheadedness, dyspnea, and constitutional symptoms—nonspecific findings that can delay diagnosis.^2^^,^^4^

Intracardiac masses are typically vegetations or thrombi, with a smaller subset including neoplasms such as myxomas, hamartomas, sarcomas, and metastases from breast, lung, or melanoma malignancies.^2^^,^^3^^,^^8^ Of the reported cases, 16 involved the right atrium, with only 2 documenting intra-atrial septum involvement. Our case demonstrates ventricular septum involvement, which has not been previously reported in BL.^8^

Traditional diagnosis of cardiac neoplasms required thoracotomy, but modern approaches include transesophageal echocardiography-guided cardiac biopsy, percutaneous intracardiac biopsy, and endomyocardial biopsy. In this case, the presence of accessible splenic lesions and an enlarged axillary lymph node allowed for minimally invasive core biopsies sufficient for diagnosis and staging. Although cardiac MRI and PET-CT remain the mainstay imaging modalities for intracardiac masses, contrast echocardiography in resource-limited settings can help characterize mass vascularity and guide decision-making.

Although BL accounts for <1% of NHL, its 24- to 48-hour doubling time means an intracardiac mass can become life-threatening within days.^2^^,^^7^ Because of atrial and ventricular septal involvement, progression or regression carries the risk of septal rupture and shunting. Other complications include pericardial invasion, arrhythmias, and heart failure.^5^, ^6^, ^7^ Reported cardiac lymphomas have described tricuspid obstruction, cardiogenic shock, and superimposed thrombus. Chemotherapy carries similar risks, including tumor lysis syndrome, coagulopathy, and arrhythmias. Tumor fragmentation can lead to obstruction of the right ventricular outflow tract or pulmonary artery.

Anthracycline cardiotoxicity may develop acutely during treatment, within the first year, or more than a year after treatment.^9^ High-risk exposure is defined as doxorubicin dose ≥250 mg/m^2^, although cardiotoxicity can occur at lower doses, particularly with cardiovascular risk factors, such as age >65 years, preexisting cardiovascular disease, diabetes, and hypertension. Baseline echocardiography with global longitudinal strain is recommended. Cancer therapy-related cardiac dysfunction is defined as left ventricular ejection fraction decline >10%-<50% and/or relative decline in global longitudinal strain >15%. Right ventricular dysfunction may also occur, though less studied.^9^ High-risk patients warrant echocardiography and biomarkers every 3 to 6 months during treatment, and moderate-to high-risk survivors at least every 5 years. Dexrazoxane remains the only Food and Drug Administration-approved agent for reducing anthracycline cardiotoxicity for cumulative doxorubicin doses of 300 mg/m^2^. Liposomal formulations reduce cardiac accumulations and offer an alternative cardioprotective strategy. Early detection with prompt initiation of heart failure therapy improves the likelihood of left ventricular ejection fraction recovery.

Cardiac lymphoma, whether primary or secondary, preferentially involves the right atrium and arises in the setting of disseminated disease, with increased risk in acquired immunodeficiency, such as HIV or transplant recipients.^1^^,^^2^ Primary cardiac lymphomas account for 2% of primary cardiac tumors and <1% of extranodal lymphomas, with the majority being diffuse large B-cell lymphoma and a smaller subset being BL.^8^ At autopsy, cardiac involvement occurs in 8.7%-20% of NHL.^1^^,^^5^ Among 94 patients with cardiac NHL, symptomatic heart failure was the most common presentation (34%), 20% were asymptomatic, and median survival was 3 months—18 vs 1 month with chemotherapy (HR: 0.16; 95% CI: 0.47-0.54; *P* = 0.0003).^10^

Cardiothoracic surgery was consulted regarding possible resection and biopsy of the intracardiac mass. The risks, however, outweighed the benefits given the disseminated disease and anticipated chemosensitivity. Reported cardiac BL cases show marked intracardiac mass reduction during induction, mirroring the resolution observed in this patient.^7^^,^^8^ Systemic therapy is therefore reasonable as initial management in chemosensitive BL with cardiac involvement, with close imaging surveillance for mechanical complications during treatment.

## Conclusions

This case illustrates a rare presentation of HIV-associated BL with cardiac involvement. The absence of cardiac symptoms in this patient—despite significant intracardiac disease—underscores the importance of early cardiac imaging in disseminated BL, regardless of presenting symptoms. BL is an aggressive malignancy with a rapid doubling time that can become life-threatening within days, necessitating expedited multidisciplinary management.

## Funding Support and Author Disclosures

The authors have reported that they have no relationships relevant to the contents of this paper to disclose.

**Take-Home Messages**

• Cardiac imaging should be pursued early in disseminated Burkitt lymphoma regardless of cardiac symptoms, as intracardiac involvement can progress from silent to life-threatening within days given the tumor’s rapid doubling time.
• Contrast-enhanced echocardiography in resource-limited settings can help characterize intracardiac masses when advanced imaging is not readily accessible.

## Appendix

Video 1 Contrast Echocardiography of the Intracardiac Mass Contrast echocardiogram demonstrating the mass attached to the interatrial septum with invasion into the crux of the heart. Contrast enhancement shows perfusion within the mass, consistent with vascularity, and helped distinguish it from thrombus or vegetation in this resource-limited setting.

Video 2 Resolution of the Intracardiac Mass Follow-up transthoracic echocardiogram approximately 2 months after initiation of chemotherapy, demonstrating resolution of the previously visualized right atrial mass and response to systemic chemotherapy.
